# Finite element analysis of modified Slongo’s external fixation in the treatment of supracondylar humeral fractures in older children

**DOI:** 10.1097/MD.0000000000037979

**Published:** 2024-05-03

**Authors:** Jingxin Zhao, Wuyi Yao, Jianxiong Ma, Bin Lu, Xinlong Ma

**Affiliations:** a Clinical College of Orthopedics, Tianjin Medical University, Tianjin, People’s Republic of China; b Trauma Department of Orthopedics, Affiliated Hospital of Chengde Medical College, Shuangqiao District, Chengde, Hebei, People’s Republic of China; c Tianjin Hospital, Tianjin, People’s Republic of China.

**Keywords:** external bracket, finite element analysis, older children, supracondylar fracture of humerus

## Abstract

Older children over 8 years old are at higher risk of elbow joint stiffness after treatment of supracondylar humeral fractures. The objective of this study was to improve the Slongo’s external fixation system for treating supracondylar humeral fractures in older children. This would be achieved by increasing fixation strength and providing a theoretical basis through finite element analysis and mechanical testing. A 13-year-old female patient with a history of previous fracture was selected for CT data processing to create a three-dimensional model of the distal humerus fracture. Two internal fixation models were established, using the Slongo’s external fixation method with Kirschner wire (Group A) and modifying the Slongo’s external fixation (Kirschner wire tail fixation) (Group B). The fracture models were then subjected to mechanical loading analysis using Finite Element Analysis Abaqus 6.14 software to simulate separation, internal rotation, and torsion loads. A PVC humeral bone model was used to create a supracondylar fracture model, and the A and B internal fixation methods were applied separately. The anterior–posterior and torsional stresses were measured using the Bose Electroforce3510 testing system, followed by a comparative analysis. The finite element simulation results showed that under the same tensile, torsion, and inversion forces, the osteotomy model fixed with Kirschner wire at the distal end in Group B exhibited smaller tensile stress and deformation compared to the unfixed osteotomy model in Group A. This indicated that the fixation strength of Group B was superior to that of Group A. According to the test results of the Bose Electroforce3510 testing system, a simple linear regression analysis was conducted using SPSS software. The K values of rotation angle-torque tests and front and rear displacement-stress tests were calculated for Groups A and B, with Group B showing higher values than Group A. The results of this study supported the significantly enhanced biomechanical reliability and stability of fracture fixation in Group B, which utilized the modified Slongo’s external fixation (Kirschner wire tail fixation). This optimized method provides a new choice for the clinical treatment of supracondylar humeral fractures in older children, backed by both clinical evidence and theoretical basis.

## 1. Introduction

Supracondylar humeral fractures are the most common type of elbow fractures in children aged 5 to 10 years.^[1,2]^ These fractures can be classified as either extension or flexion fractures based on the mechanism of injury and the displacement of the distal fracture. The most common cause of these fractures is hyperextension of the elbow, resulting in an extension fracture.^[3]^ Older children, who are over 8 years old, have biological characteristics that are more similar to adults, although they are not quite adults yet. Their healing rate is slower than younger children but faster than adults.^[4]^ Additionally, younger children may have larger fracture injuries and more severe fracture displacement, leading to a higher risk of elbow dysfunction and slower recovery of elbow function after injury.^[5]^ As a result, stable fixation is crucial for this age group, as they exhibit characteristics that are unique, being closer to adults while still having skeletal characteristics of children.^[6]^ In the case of supracondylar humeral fractures in older children, open reduction and plate internal fixation, which is commonly used for adult humeral fractures, is often not recommended.^[7–9]^ Instead, the Slongo’s external fixation provides a new treatment idea.^[10]^ This method preserves the distal humerus growth plate and offers better fixation strength compared to Kirschner wire treatment, allowing for early functional exercise. This study aimed to optimize this treatment method, enhancing fracture fixation strength without increasing the number of internal fixations, thereby facilitating early postoperative functional exercise.^[11]^ The theoretical basis for the clinical treatment of supracondylar humeral fractures in older children was established through finite element analysis and the use of the Bose Electroforce3510 test system simulation analysis.

## 2. Materials and methods

### 2.1. Finite element analysis

#### 2.1.1. Establishment of supracondylar fracture model of humerus

**Figure 1.**
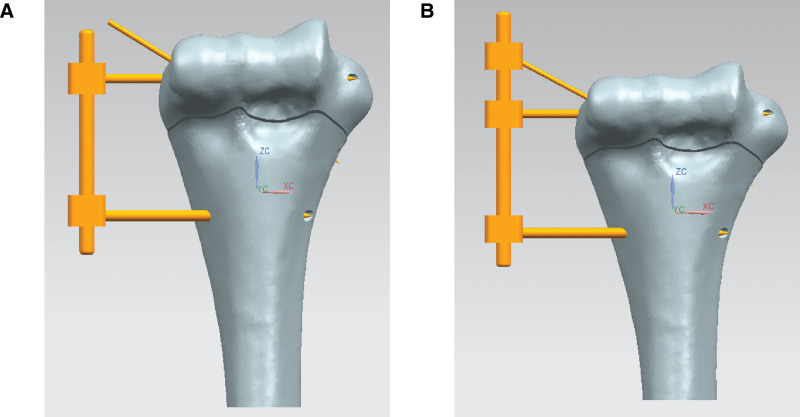
Slongo’s external fixation and Kirschner wire implantation model. (A) Kirschner wire tail is not fixed; (B) Kirschner wire tail is fixed.

Normal 3D CT of the distal humerus of a 13-year-old girl was selected from the PACS system of hospital images, and the image file (DICOM format) was imported into minics image processing software to obtain the 3D model of the distal humerus of children. The 3D model of the distal humerus of children was smooth processed with Geomagic Studio 2012. The finite element preprocessing software Hypermesh 13.0 was used for the finite element mesh division of the distal humerus model and material assignment. The three-dimensional modeling software Solidworks 2012 was used to simulate supracondylar humeral fractures in children, which were the most common extended fractures with 30 degrees sagittal plane across the intercondylar fossa in the clinic, and then the osteotomy surface was established, the model was divided by the osteotomy surface, and finally the external frame and Krantler needle were implanted into the humerus, as shown in Figure 1.

#### 2.1.2. Material properties

The humerus model material was set as the outer cortical bone, the inner cancellous bone, and the material of the Kirschner wire and the external fixation was set as stainless steel. Table 1 shows the material properties.

**Table 1:** Material property information.

	Cortical bone	Cancellous bone	K-wire	External fixation
Density ρ (kg/m^−3^)	1640	320	7930	7930
Modulus of elasticity E (GPa)	16.7	16	210	210
Poisson ratio ε	0.3	0.3	0.3	0.3

#### 2.1.3. Contact settings

The friction contact between Kirschner needle and bone needle and humerus is set, the friction coefficient is 0.2; the friction contact between the osteotomy surfaces is set, and the friction coefficient is 0.2; the bonding contact is arranged between the Kirschner wire and the bone needle and the connecting rod of the external fixation.

#### 2.1.4. Mesh division

Table 2 shows the material properties.

**Table 2:** Material properties.

Serial number	Humerus mesh size	K-wire mesh size	External fixation mesh size	Number of nodes	Number of units
a	1.5 mm	0.5 mm	0.5 mm	466,516	319,268
b	1.5 mm	0.5 mm	0.5 mm	552,815	379,935

#### 2.1.5. Setting boundary conditions

**Figure 2.**
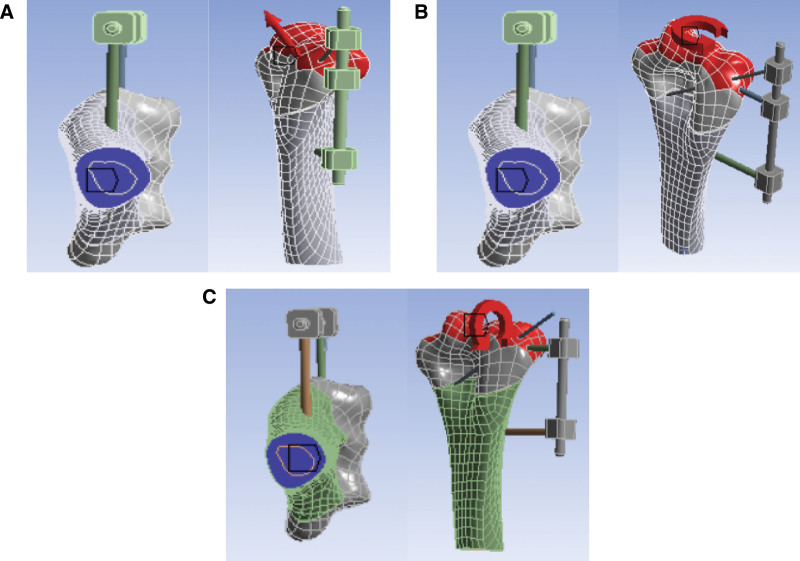
Boundary condition diagram. (A) Diagram of stretching boundary conditions; (B) schematic of the torsional boundary conditions; (C) diagram of inversion boundary conditions.

Stretching: In the normal direction along the osteotomy surface, 30N tension is applied to the distal end of the humerus, while the upper section of the humerus is fixed. Please refer to Figure 2A.

*Torsional*: Along the normal direction of the upper section, a torque of 135 Nm m is applied to the distal end of the humerus, while the upper section of the humerus is fixed. The direction of clockwise torsion is observed from the upper side of the osteotomy. Please refer to Figure 2B.

*Inversion*: Along the normal direction of the sagittal plane, a torque of 100 N mm is applied to the distal end of the humerus, while the upper section of the humerus is fixed. The direction of counterclockwise twisting is observed from the medial side of the distal humerus to the lateral side. Please refer to Figure 2C.

### 2.2. Bose Electroforce3510 test

#### 2.2.1. Model making

The humeral supracondylar fracture model of the humerus was made by transverse amputation of the humerus intercondylar fossa with imported PVC material (Fig. 3A). Two internal fixation methods were adopted, Group A and Group B, respectively.

**Fnigure 3.**
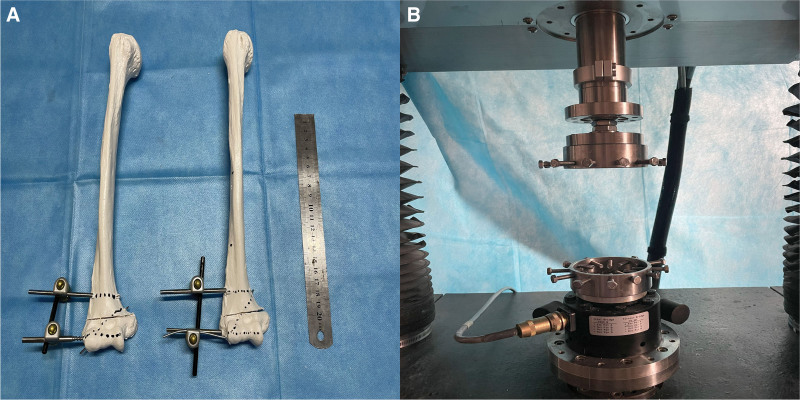
Fracture model and Bose Electroforce3510 test system.

The Bose Electroforce3510 test system (Fig. 3B) was used to mix the 2 ends of the fixed model with denture base resin II. The speed was controlled at 0.167 mm/s, the maximum displacement was 15 mm, and the stress unit was N and the displacement unit was mm. The front and rear displacement-stress tests were performed on 2 different internal fixation models.

Similarly, the Bose Electroforce3510 test system was used to perform rotation angle-torque tests on 2 different internal fixation models of supracondylar humeral fractures, using denture base resin II hybrid fixed model with rotation speed controlled at 0.1 degrees/s, rotation Angle unit in degrees and torque unit in N · m.

## 3. Results

### 3.1. Results of finite element analysis

1. The analysis of tensile strength simulation results is shown in Table 3.
2. With the external fixation (K-wire tail is not fixed)—tensile strength simulation analysis cloud image.

**Table 3:** Tensile strength analysis results.

Fixed type	Humeral stress (MPa)	Deformation of the humeral (mm)	External fixation stress (MPa)	Deformation of the external fixation (mm)
Group B	126.04	1.776	620.1	1.815
Group A	281.4	1.872	715.52	1.251

**Figure 4.**
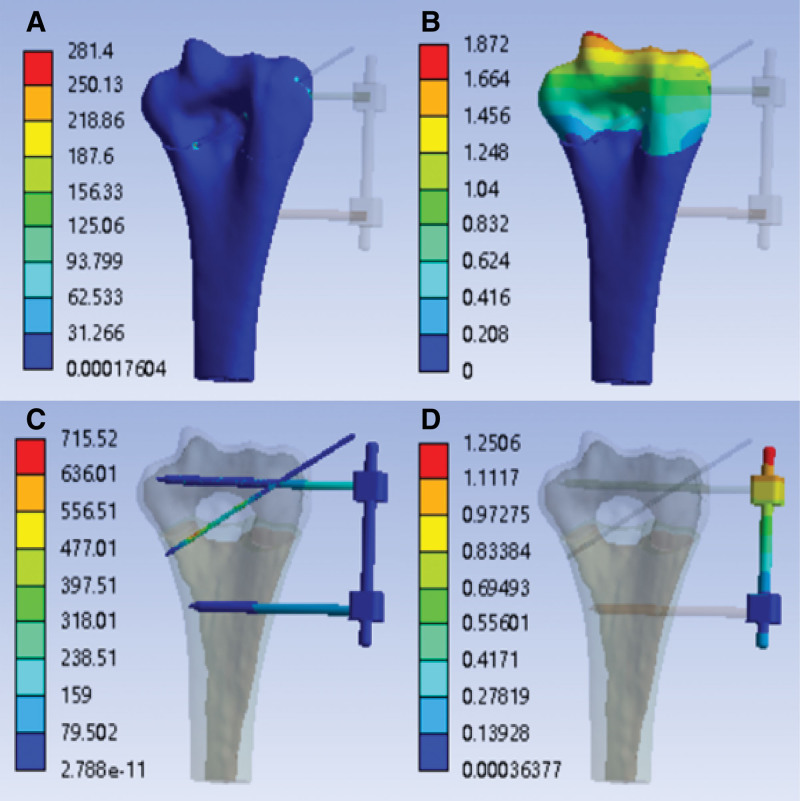
With the external fixation (K-wire tail is not fixed)—tensile strength simulation analysis cloud image. (A) Stress cloud image of humerus (MPa); (B) humerus deformable cloud image (mm); (C) stress cloud image of external fixation (MPa); (D) external fixation deformable cloud image (mm).

**Figure 5.**
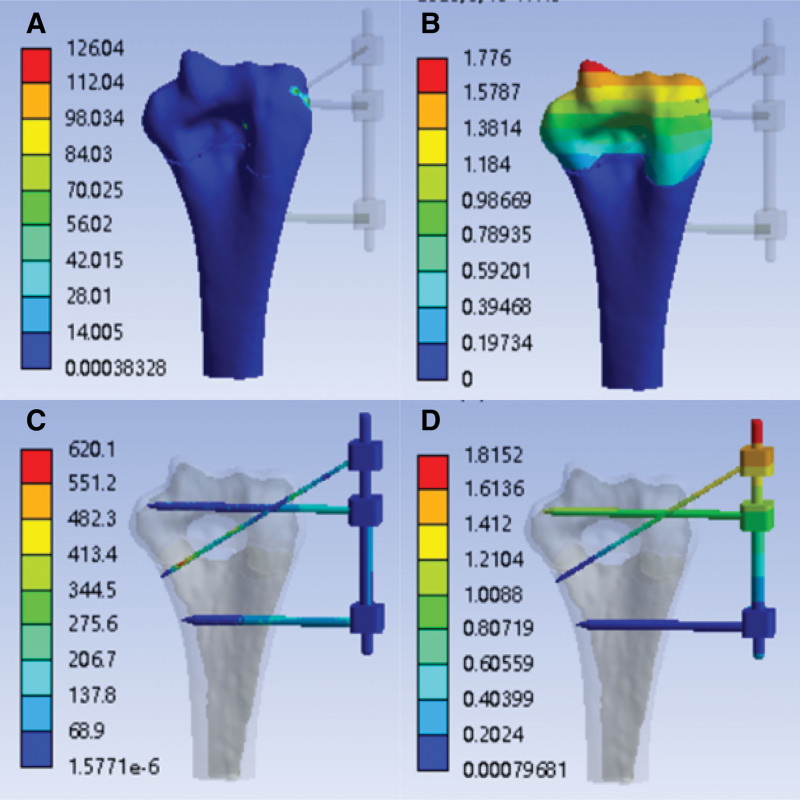
With the external fixation (K-wire tail is fixed)—tensile strength simulation analysis cloud image. (A) Stress cloud image of humerus (MPa); (B) humerus deformable cloud image (mm); (C) stress cloud image of external fixation (MPa); (D) external fixation deformable cloud image (mm).

After finite element simulation, the finite element analysis cloud diagram of tensile strength with external fixation (K-wire tail is not fixed) is obtained, as shown in Figure 4A–D:

With the external fixation (K-wire tail is fixed)—tensile strength simulation analysis cloud image After finite element simulation, the finite element analysis cloud diagram of tensile strength with external fixation (K-wire tail is fixed) is obtained, as shown in Figure 5A–D:

1. The simulation results of torsional strength are shown in Table 4:
2. With the external fixation (K-wire tail is not fixed)—torsional strength analysis cloud image.

**Table 4:** Torsional strength analysis results.

Fixed type	Humeral stress (MPa)	Deformation of the humeral (mm)	External fixation stress (MPa)	Deformation of the external fixation (mm)
Group B	126.43	0.181	386.19	0.250
Group A	175.78	0.194	352.8	0.230

**Figure 6.**
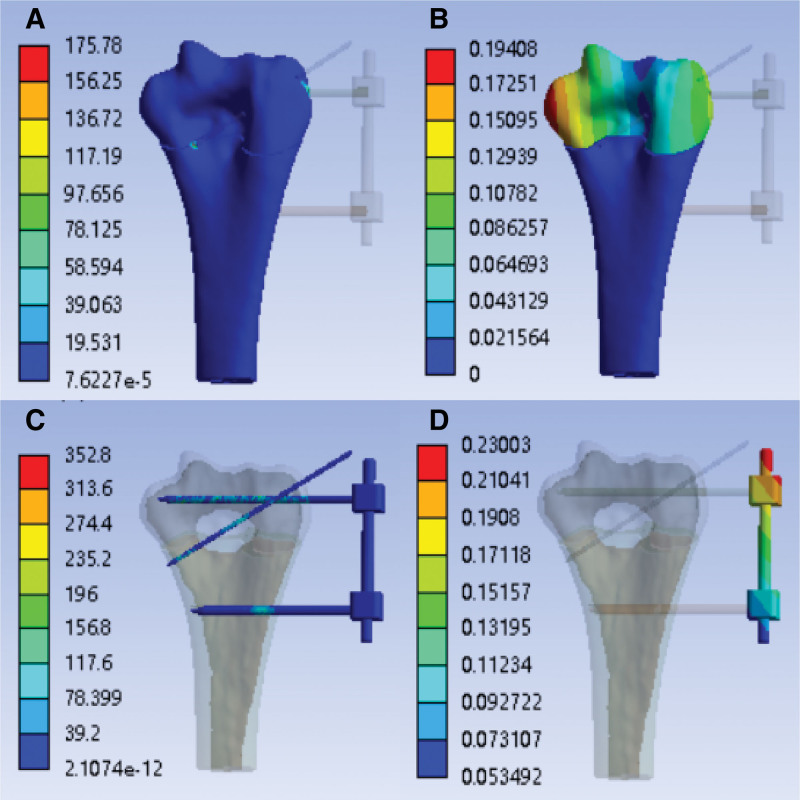
With the external fixation (K-wire tail is not fixed)—torsional strength analysis cloud image. (A) Stress cloud image of humerus (MPa); (B) humerus deformable cloud image (mm); (C) stress cloud image of external fixation (MPa); (D) external fixation deformable cloud image (mm).

After finite element simulation, the finite element analysis cloud diagram of torsional strength with external fixation (the tail end is not fixed) is obtained, as shown in Figure 6A–D:

With the external fixation (K-wire tail is fixed)—torsional strength analysis cloud image.

**Figure 7.**
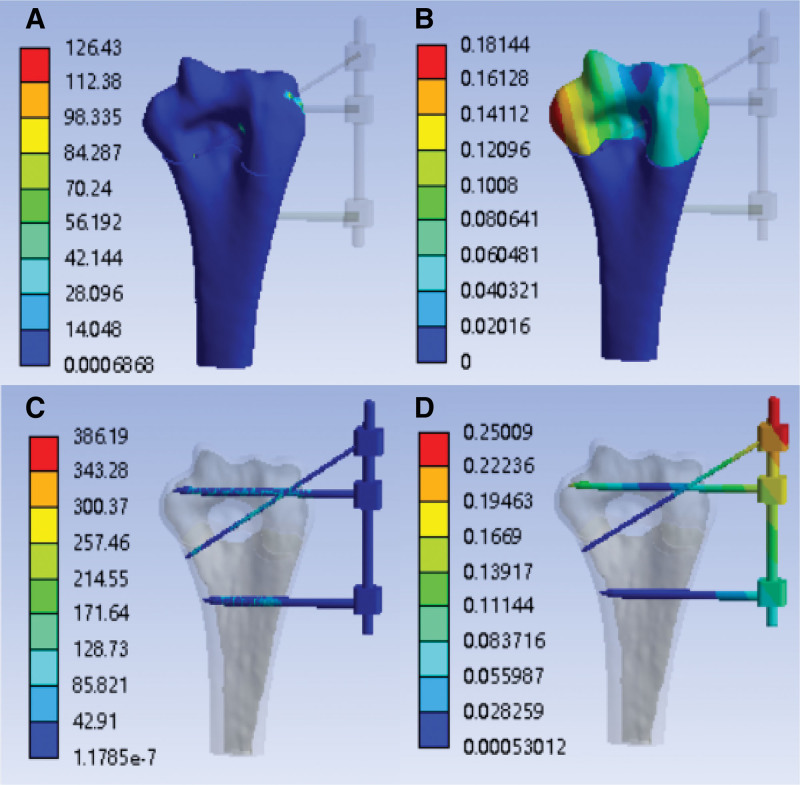
With the external fixation (K-wire tail is fixed)—torsional strength analysis cloud image. (A) Stress cloud image of humerus (MPa); (B) humerus deformable cloud image (mm); (C) stress cloud image of external fixation (MPa); (D) external fixation deformable cloud image (mm).

After finite element simulation, the finite element analysis cloud diagram of torsional strength with external fixation (fixed end) is obtained, as shown in Figure 7A–D:

1. The simulation analysis results of inversion strength are shown in Table 5:
2. With the external fixation (K-wire tail is not fixed)—inversion strength analysis cloud image.

**Table 5:** Inversion strength analysis results.

Fixed type	Humeral stress (MPa)	Deformation of the humeral (mm)	External fixation stress (MPa)	Deformation of the external fixation (mm)
Group B	129.84	0.305	386.66	0.317
Group A	175.61	0.366	352.78	0.221

**Figure 8.**
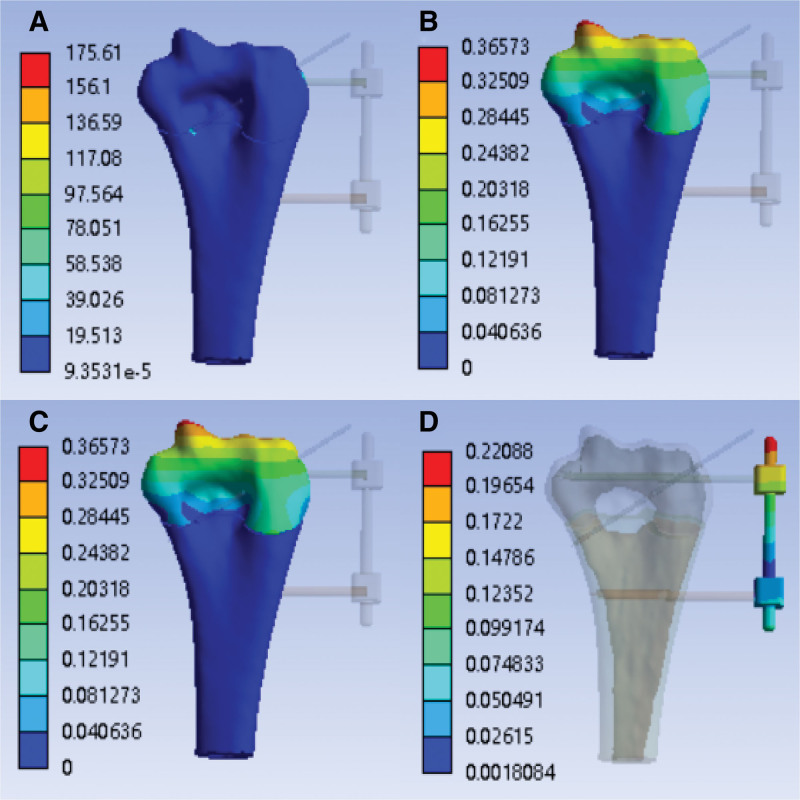
With the external fixation (K-wire tail is not fixed)—inversion strength analysis cloud image. (A) Stress cloud image of humerus (MPa); (B) humerus deformable cloud image (mm); (C) stress cloud image of external fixation (MPa); (D) external fixation deformable cloud image (mm).

After finite element simulation, the finite element analysis cloud diagram of the inversion strength with the external fixation (K-wire tail is not fixed) is obtained, as shown in Figure 8A–D:

With external fixation (K-wire tail is fixed)—inversion strength analysis cloud image.

**Figure 9.**
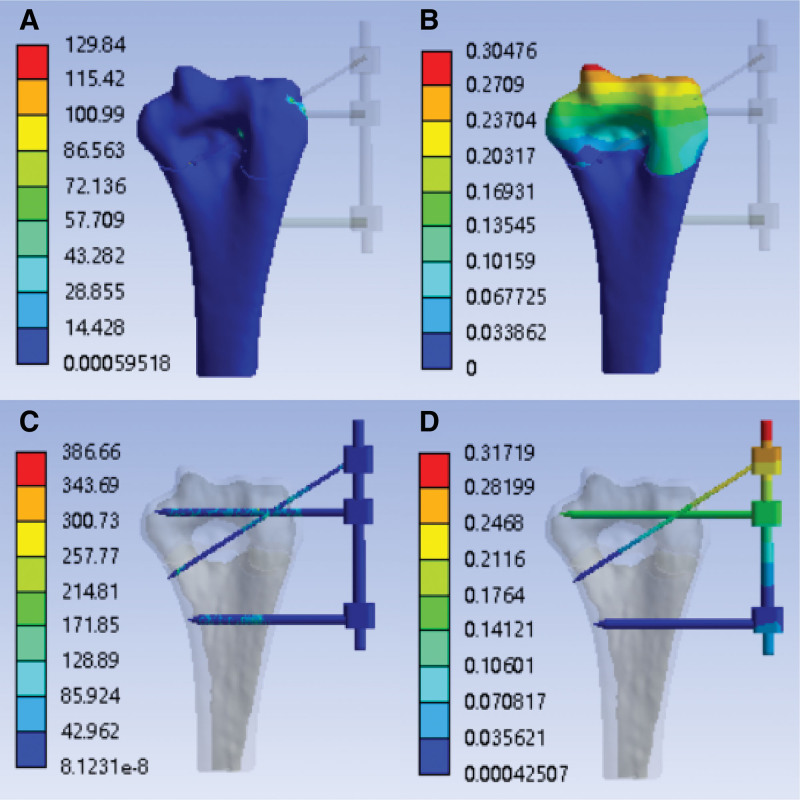
With the external fixation (K-wire tail is fixed)—inversion strength analysis cloud image. (A) Stress cloud image of humerus (MPa); (B) humerus deformable cloud image (mm); (C) stress cloud image of external fixation (MPa); (D) external fixation deformable cloud image (mm).

After the finite element simulation, the finite element analysis cloud diagram of the overturning strength with the external fixation (K-wire tail is fixed) is obtained, as shown in Figure 9A–D:

Based on the Finite Element Analysis, under the application of the same direction of force and fixation constraints, a comparison of the 2 fixation methods was conducted using the simulation results from Tables 3, 4, and 5. The results revealed that the modified Slongo’s external fixation with Kirschner wire fixation at the distal end (Group B) exhibited lower levels of tensile stress, torsional stress, internal rotation stress, and deformation compared to the Slongo’s external fixation (Group A) in the osteotomy model. Therefore, the fixation approach in Group B is more stable and preferable.

### 3.2. Test results of Bose Electroforce3510

GraghPad software was used to draw the point and connection diagram for each group of tests, and spss software was used to analyze the simple linear regression model. The relationship between the displacement, torque and rotation angle before and after the stress and fracture models was statistically significant in the 2 groups of tests. The K value was calculated, and group B was greater than group A, so the scheme of group B was more stable and better. As shown in Table 6, *P* < .001 for each group.

**Table 6:** Comparison of k value of test results between Group A and Group B.

Fixed type	Group A (“slongo’s” external fixation + Kirsch wire)	GroupB “slongo’s” external fixation (with K-wire tail is fixed)
Forward and backward displacement—stress test K value	73.434	79.766
Rotational angle-torque test K value	0.831	0.882

## 4. Discussion

With the advancement in computer processing power and numerical computing techniques, finite element analysis has become a valuable tool in the field of orthopedics, including pediatric orthopedics.^[12–14]^ By utilizing finite element calculations, it is possible to analyze the distribution of stress, strain, and displacement in the fixation of supracondylar humeral fractures, thereby facilitating improvements and optimizations in design.^[15,16]^ Moreover, in addition to meeting the mechanical performance requirements of medical devices, it is crucial to adhere to the principles of fracture fixation. These principles include: (1) providing robust internal fixation that fulfills the biomechanical needs, (2) employing minimally invasive surgical techniques, and (3) enabling early pain-free active joint movement. By considering these factors, the aim is to meet the clinical requirements and ensure successful clinical applications.

The results revealed that the modified Slongo’s external fixation with Kirschner wire fixation at the distal end (Group B) exhibited lower levels of tensile stress (126.04 Mpa < 281.4 MPa), torsional stress (126.43 Mpa < 175.78 MPa), internal rotation stress (129.84 MPa < 175.61 MPa) and deformation compared to the Slongo’s external fixation (Group A) in the osteotomy model. Therefore, the fixation approach in Group B is more stable and preferable. In recent years, biomechanical studies have demonstrated that the Slongo’s external fixation offers superior stability under load compared to cross Kirschner wire. As a result, it has been proven to be an effective method for treating supracondylar humeral fractures in older children.^[10,17]^ This technique involves the placement of a Schanz pin at each end of the fracture. The distal Schanz pin is carefully positioned above the lateral epicondyle of the humeral condyle, coinciding with the rotation center of the elbow joint, to prevent any damage to the growth plate of the lateral condyle. On the other hand, the proximal Schanz pin is inserted transversely through a small incision directly into the humerus, passing through both layers of the cortex. To provide additional fixation and reinforce rotational stability, an obliquely placed Kirschner wire is inserted through the fracture line on the lateral side. Furthermore, the Schanz pins can aid in fracture reduction.^[18]^ This surgical technique is relatively safe and straightforward, requiring fewer intraoperative fluoroscopy sessions. Importantly, postoperatively, the external fixator enables early mobilization of the elbow joint, facilitating a quicker restoration of normal function in pediatric patients.

Based on previous clinical experience, the use of smooth Kirschner wires poses a risk of wire migration and accidental dislodgement during early elbow joint functional exercises.^[19,20]^ This can result in internal fixation failure, decreased fixation strength, and subsequent fracture displacement. To address this issue, Group B introduced a new fixation method whereby the distal end of the Kirschner wire is secured to the external fixation connecting rod. This integration creates a comprehensive external fixation system that effectively prevents wire migration or movement. In this study, simulation analysis using finite element analysis and the Bose Electroforce3510 testing system was conducted to compare the 2 fixation methods. The finite element analysis evaluated stress and deformation in the humerus, as well as stress and deformation in the external fixator, under the same force direction and fixation constraints. The findings revealed that Group B, with the Kirschner wire fixed to the external fixation connecting rod, exhibited superior fixation effectiveness. The results from the Bose Electroforce3510 testing system supported this conclusion. The new fixation method offers a novel approach for treating supracondylar humeral fractures in older children. It not only avoids the need for additional internal fixation devices but also overcomes the limitations associated with smooth Kirschner wire fixation, thereby advancing existing treatment techniques. Although supracondylar humeral fractures in older children are relatively rare,^[21]^ the clinical application of Group A, which involves Kirschner wire-assisted Slongo external fixation, has shown positive treatment outcomes, facilitating prompt recovery of normal function. Building upon this experience, the fixation method has been improved and optimized, as demonstrated by the significant enhancements observed in all aspects of Group B fixation.

In conclusion, the modified Slongo’s external fixation system fixation method is a recommended approach for the treatment of supracondylar humeral fractures in older children. This study provides significant theoretical support for its effectiveness. However, additional clinical research is necessary to validate these findings.

## Acknowledgments

We thank all of the patients involved in the study.

## Author contributions

**Investigation:** Jianxiong Ma.

**Validation:** Bin Lu.

**Writing – original draft:** Jingxin Zhao, Wuyi Yao.

**Writing – review & editing:** Xinlong Ma.
